# Kidney Cancer and Potential Use of Urinary Extracellular Vesicles

**DOI:** 10.3389/or.2024.1410450

**Published:** 2024-05-23

**Authors:** Linh Nguy-Hoang Le, Javaria Munir, Eun-Bit Kim, Seongho Ryu

**Affiliations:** ^1^ Department of Integrated Biomedical Science, Soonchunhyang University, Cheonan, Republic of Korea; ^2^ Soonchunhyang Institute of Med-Bio Science (SIMS), Soonchunhyang University, Cheonan, Republic of Korea; ^3^ Department of Nutrition and Health Sciences, University of Nebraska-Lincoln, Lincoln, NE, United States

**Keywords:** urine, extracellular vesicle, exosome, kidney cancer, renal cell carcinoma, biomarker

## Abstract

Kidney cancer is the 14th most common cancer globally. The 5-year relative survival rate of kidney cancer at a localized stage is 92.9% and it declines to 17.4% in metastatic stage. Currently, the most accurate method of its diagnosis is tissue biopsy. However, the invasive and costly nature of biopsies makes it undesirable in many patients. Therefore, novel biomarkers for diagnosis and prognosis should be explored. Urinary extracellular vesicles (uEVs) are small vesicles (50–200 nm) in urine carrying nucleic acids, proteins and lipids as their cargos. These uEVs’ cargos can provide non-invasive alternative to monitor kidney health. In this review, we have summarized recent studies investigating potential use of uEVs’ cargos as biomarkers in kidney cancer for diagnosis, prognosis and therapeutic intervention.

## Introduction

Kidney cancer is the 14th most common cancer globally and one of the top ten most common cancers in males. According to the GLOBOCAN 2020, there are 431,288 new cases and 179,386 new deaths of kidney cancer. Incidence and mortality rate of kidney cancer are 6.1 and 2.5 in males, and 3.2 and 1.2 in females, respectively [1]. Notably, the 5-year relative survival rate of kidney cancer at localized stage is 92.9%, and sharply decline to 17.4% in metastatic stage [2]. Different types of kidney cancer are classified based on histology and require different targeting therapies. Therefore, novel biomarkers for diagnosis and prognosis should be investigated to ameliorate the survival rate of kidney cancer.

Extracellular vesicles (EVs) are the lipid bilayer membrane-bound particles which contain abundant biological information (nucleic acids, proteins, metabolites and lipids). They have various sizes including exosomes (50–200 nm), ectosomes (100–1,000 nm) and apoptotic bodies (50–5,000 nm). Body fluids such as blood, plasma, serum and urine, and cell culture media are the great resources of EVs [3]. Recent studies have reported that EVs can be taken up from donor cells to recipient cells and considered as a new tool for intercellular communication [4]. Due to the membrane of EVs, their cargos are protected from degradation by proteases and other enzymes. This protection of cargos enables them to be delivered to the recipient cell or organ. Plasma-derived exosomal protein profiles exhibit unique patterns of cargos that allow us to classify primary tumors. These unique patterns of plasma-derived EVs may be utilized to predict the tumor origin in patients [5]. Furthermore, EVs contain biomarkers for predicting future site of metastasis. Therapeutic approaches can be targeting EVs and inhibiting specific organ uptake, targeting EVs-induced changes in future site of metastasis, and using EVs as drug delivery system [6]. The emerging potential role of EVs in diagnosis and therapy with highest sensitivity has led to increased interest in their investigation.

## Extracellular Vesicles (EVs) in Kidney Cancer

Renal cell carcinoma (RCC) is the most common type of kidney cancer in adults. It ranks as the third most common urological cancer following prostate and bladder cancer. RCC starts in the renal tubules that clean the blood and produce urine. In addition, RCC in the later stages disseminates to other organs frequently, i.e., bones, lungs, or brain. Histopathologically, the most common subtypes of RCC are: clear cell (75%–85%), papillary (10%–15%), and chromophobe (5%–10%) renal cell carcinoma. Clear cell renal cell carcinoma (ccRCC) has the lowest survival rate among these prevalent subtypes [7]. The common metastatic sites of ccRCC are lungs (54%), bone (18%), lymph nodes (16%) and liver (6%) [8].

Kidney surgery is the gold intervention to manage localized kidney cancer. This includes partial nephrectomy that removes only the cancerous portion of the kidney, while radical nephrectomy removes the entire kidney [9]. Further treatments for kidney cancer comprise of radiation therapy, chemotherapy, targeting medicines, cryoablation, radiofrequency ablation and microwave ablation [10–12]. Moreover, in an effort to gain insight into targeting therapy, engineering EVs show potentially effective vehicles against RCC. TRAIL (TNF-related apoptosis-inducing ligand) engineered MSC-derived EVs showed a significant effect on TRAIL resistant renal cancer cell lines, e.g., RCC10 and HA7-RCC [13]. Mesenchymal stem cell-derived EVs have mild effect on renal cancer by enhanced apoptosis and preventing proliferation [14]. Currently, diagnosis of kidney cancer is composed of physical exam, urine test, blood test, intravenous pyelogram, CT scan, ultrasound test and biopsy [9]. RCC raises a big concern due to high metastatic rate, mortality rate, increased incidence and therapeutic resistance. Diagnosing solid tumor becomes challenging in circumstances of unconventional tumor cell patterns or limited tissue samples [15].

Several pioneer studies have shown the potential of EVs in RCC diagnosis. Remarkable markers CA9, CD70 and CD147, which are expressed in ccRCC tumor tissues, are also identified in secreted EVs. Expression of these proteins in EVs validate their origin from the primary kidney tissue and can be the reliable biomarkers for less invasive and tumor-specific diagnostic methods [16]. Cargos of EVs derived from clear cell RCC, papillary RCC (pRCC) and benign kidney cell lines have unique signatures, thereby they can be used to discriminate not only RCC subtype, but also RCC from benign renal cells. Twenty and thirty-four exosomal proteins are exclusively enriched in EVs released from ccRCC and pRCC, respectively. Exosomal mRNA of EPCAM, PRKCZ, PXDN, CXADR, EPS8L1, HOXA7, LAD1, MYO1D, ROCK2, and SLC35A3 are unique to EVs of benign renal cell, but not ccRCC [17]. In contrast, an epithelial tumor cell marker EpCAM heterogeneously expresses in both normal tubular and ccRCC samples [16]. Moreover, CDH2, COL7A1, FGFR2, BMPR1B, HDHD3, ICAM1, KIAA1462, and PFKFB4 mRNA are found only in ccRCC-derived EVs [17].

Besides proteins and mRNAs, non-coding RNAs, e.g., microRNAs (miRNAs), long non-coding RNAs (lncRNAs) and circular RNAs are abundantly enriched in EVs. miR-205 is significantly downregulated in EVs secreted by ccRCC cell lines 786-O compared to normal cells HK-2 [18]. Many studies have disclosed the tumor suppressive role of miR-205 in renal cancer. miR-205 inhibits Src-mediated oncogenic pathway, negatively regulates EMT transcription factor ZEB2, suppresses PTEN/AKT pathway, and downregulates VEGFA and PI3K/Akt/mTOR signaling [19–22].

Examining the miRNA profiling of plasma derived-EVs from RCC patients exhibits upregulated expression of miR-149-3p and miR-424-3p, and downregulated miR-92a-1-5p expression. These miRNAs are potential diagnostic biomarkers for RCC with area under the curve (AUC), the sensitivity and specificity of hsa-miR-92a-1-5p (0.8324, 87.5% and 77.3%), hsa-miR-149-3p (0.7188, 75% and 72.7%) and hsa-miR-424-3p (0.7727, 75% and 81.8%), respectively [23]. The mechanism how these dysregulated miRNAs are involved in tumor progression needs to be investigated.

Moreover, exosomal miR-210 is upregulated in ccRCC patients compared to healthy control, especially, the high expression of this miRNA is significantly associated with patients at T3/T4 tumor stage, Fuhrman grade III/IV and metastasis [24]. In addition, exosomal miR-210 is significantly elevated in renal cell lines HK-2, 786-O, and SN12-PM6 upon hypoxic condition induced by CoCl_2_. miR-210 has proven to be a good prognostic biomarker to monitor recurrence after primary tumor resection as well. Indeed, miR-210-3p, which is upregulated in RCC tissue, has high level in serum and urine of RCC patients, and significantly decreases in post-operative patients’ urine within a month [24–26]. Nakada et al. have demonstrated that HIF1α protein accumulation induces miR-210 expression, which subsequently suppresses E2F transcription factor 3 and causes centrosome amplification and aneuploidy in ccRCC cell lines [27]. Another study also showed that miR-210 silencing in metastatic RCC cells deregulates the HIF1α protein [28]. Furthermore, miR-210-5p is a downstream target of exosomal circular RNA_400068 which is isolated from Caki-1 and Caki-2 cell derived-EVs (ccRCC cell lines) and plays a role as tumor suppressor in RCC [29].

Long non-coding RNAs such as exosomal lncARSR and lncRNA IGFL2-AS1 facilitate sunitinib resistance in RCC cells. Both of these lncRNAs also transform sunitinib-sensitive cells into resistant cells. Hence, EVs are the effective delivery package that disseminate drug resistance in advanced RCC. These lncRNAs might be prognostic indicators and potential therapeutic approach in chemotherapeutic resistance [30, 31].

## Urinary Extracellular Vesicles (uEVs) in Kidney Cancer

Urinary extracellular vesicles (uEVs), which originate from bladder, prostate and kidney, have gained immense investigation since uEVs reflect the pathology of the kidney [32, 33]. First and mid-stream urine are collected as an appropriate resource for EV analysis [34]. uEVs are isolated by several methods such as ultracentrifugation, chemical precipitation, size exclusion chromatography and ultrafiltration [35]. Tamm Horfall protein (THP) is abundant in urine and can trap uEVs. Detergents such as Dithiothreitol (DTT)/urea, which are used for entrapping EVs, enhance yield of uEVs [36, 37].

Transmission electron microscopy (TEM) and nanoparticle tracking analysis (NTA) are utilized to identify the morphology and size of EVs. NTA is preferable to determine the size distribution of EVs than TEM, because EVs usually coagulate and form a bundle on the coal-copper grid [16]. EV markers are characterized by immune blotting. CD63 is found to be a representative exosomal marker for RCC cell lines, e.g., 786-O, 769-P, ACHN, Caki-2, Caki-1 and RCC53 due to its stable expression rather than other exosomal markers CD9 and CD81, which have variable expression among RCC cell lines [16]. Thus, anti-CD63 nanobodies have been applied for an efficient isolation of EVs from urine with high purification [38]. CANX is identified as a negative EV marker for RCC cell lines by spatial proteomics analysis [16, 17]. Indeed, human renal cancer tissue derived-EVs are enriched in CD63, CD81 and flotillin-1 [3]. Notably, clinical urine samples also contain bacteria. These bacteria have been known as a resource of bacterial EVs and can interfere with the analysis results. Furthermore, bacterial EVs induce the cytokine secretion of renal cells [39]. This implies that the consideration about storage urine samples should be rigorously considered.

In kidney cancer, examining uEVs is a non-invasive method than tissue biopsy and longitudinal monitoring to observe the condition of the disease (Table 1). The contents of uEVs are also identified in the tissue of origin [16]. Studies have shown that compared to serum miRNAs, urinary miRNAs provided a stronger signature for acute kidney injury by oxalic acid poisoning [48]. Secreted EVs are comparable in human urine and various immortalized human kidney cell lines, e.g., podocyte, glomerular endothelial, mesangial and proximal tubular cells. This suggests that *in vitro* experiments may imitate the *in vivo* condition [49].

**TABLE 1:** Studies of urinary extracellular vesicles’ cargos in kidney cancer.

Types of uEVs’ cargos			Sample size	Profiling methods	Differential expression or modulation^a^	ROC curve analysis^b^ (AUC, sensitivity, specificity)	Application	References
Nucleic acids	mRNA	GSTA1, CEBPA, PCBD1	33 Kidney cancer vs. 22 healthy controls	Microarray, RT-qPCR	Downregulated	N/A	Discriminate patients of low-Fuhrman-grade ccRCC from healthy controls and non-ccRCC	[34]
		ALOX5, RBL2, VEGFA, TLK2	11 Tumor tissue and tumor adjacent tissue	nCounter PanCancer Progression Panel (NanoString Technologies)	Upregulated	N/A	RCC-specific urine EV biomarkers	[40]
		NME2, AAMP, CAPNS1, VAMP8, MYL12B	4 ccRCC patients vs. 6 healthy controls	Ion Torrent PGM sequencing platform (Thermo Fisher Scientific)	Upregulated	N/A	Specific for stage I ccRCC	[41]
	miRNA	miR-126-3p and miR-449a	81 ccRCC patients vs. 33 healthy controls	RT-qPCR	Upregulated	0.84, 83.8%, 62.5%	Preferable discrimination by combination of miRNAs	[4]
		miR-126, miR-145	46 type 2 diabetic patients	RT-qPCR	Upregulated	N/A	Induce EMT	[42]
		miR-204-5p	10Xp11 tRCC model mice vs. 10 controls	miRCURY LNA miRNA Custom PCR Panels (Exiqon; Qiagen, Hilden, Germany)	Upregulated	N/A	Biomarker for early detection of Xp11 tRCC	[43]
		miR-210-3p	38 ccRCC patients vs. 10 healthy controls	RT-qPCR	Upregulated	N/A	Potential non-invasive biomarker for diagnosis and surveillance after nephrectomy or treatment responses	[25]
		miR-224-5p	6 RCC patients vs. 6 healthy controls	RT-qPCR	Upregulated	N/A	Biomarkers to predict PD-1/PD-L1 treatment responses	[44]
		miR-30c-5p	42 ccRCC patients vs. 30 healthy donors	Next-generation sequencing	Downregulated	0.8192, 68.57%, 100%	Specific biomarker for RCC	[45]
				RT-qPCR				
Proteins		CAIX	29 RCC patients vs. 23 healthy controls	Proxeon EasynLC System (Proxeon Biosystems) MaXis hybrid UHR-QToF system (Bruker Daltonics)	Upregulated	0.862 ± 0.054, N/A, N/A	uEV proteome involved in the RCC pathogenesis or progression	[46]
		CP				1, N/A, N/A		
		MMP9				0.938 ± 0.035, N/A, N/A		
		PODXL				1, N/A, N/A		
		DKK4				0.979 ± 0.025, N/A, N/A		
		CD10			Downregulated	0.794 ± 0.083, N/A, N/A		[46]
		DPEP1				0.760 ± 0.083, N/A, N/A		
		EMMPRIN				0.879 ± 0.060, N/A, N/A		
		Syntenin 1				0.733 ± 0.089, N/A, N/A		
		AQP1				0.891 ± 0.050, N/A, N/A		
Lipids		Phosphatidylinositol phosphate, Lyso-phospholipids, Phosphatidylethanolamines, Phosphatidylcholines, Mono/Di/Tri-glycerols, Phosphatidic acids, Gangliosides, Prostanoids	8 ccRCC patients vs. 8 healthy controls	CapLC system (Waters)	Modulation	N/A	Lipid composition of uEVs involved in RCC	[47]
				Q-TOF-Ultima in-strument (Micromass, Waters)				

N/A, not available.

^a^ Differential expression or modulation of the cargos in uEVs of kidney cancer compared to healthy controls.

^b^ Receiver Operating Characteristic (ROC) curve.

Exosomal miRNAs from urine can differentiate RCC from healthy individuals. uEVs’ miR-126-3p is significantly plummeted in ccRCC patients compared to healthy controls (AUC: 0.74; 95% CI, 0.5948–0.8880; *p* = 0.004) [4]. The low expression of miR-126, caused by lncRNA DUXAP8, intensely associated with poor survival rate and metastatic RCC [50]. Tumor suppressor miR-126 can eradicate RCC progression via either SLC7A5 and SEPRINE1/mTOR/HIF pathway or EGFL7/ERK/STAT3 axis [51, 52]. Remarkably, combining urinary miR-126-3p and miR-449a is feasible for a sensitive distinction between ccRCC and healthy individuals, namely, AUC: 0.84; 95% CI, 0.7620–0.9151; *p* < 0.0001, the specificity and sensitivity are 83.8% and 62.5%, respectively. After nephrectomy, these miRNA levels recover comparable expression of healthy samples [4].

Additionally, lncARSR enhances sunitinib resistance by competitively binding miR-34 and miR-449 which facilitates upregulation of AXL/c-MET and the activation of STAT3, AKT, and ERK signaling in resistant RCC cells [31]. The low levels of exosomal shuttle RNAs consisting of GSTA1, CEBPA and PCBD1 in ccRCC patients relative to the healthy controls, are well defined in ccRCC, while these three genes have high expression in non ccRCC. One month after nephrectomy in ccRCC patients, these exosomal shuttle RNA levels are recovered [34].

Kuczler et al. initially carried out a comparative study of exosomal mRNA in urine and tissue of RCC samples. Exosomal mRNA transcripts of ALOX5, RBL2, VEGFA, TLK2 are specifically identified in tissue and uEVs of ccRCC patients [40]. Furthermore, uEV-derived mRNA transcripts of NME2, AAMP, CAPNS1, VAMP8, and MYL12B are significantly downregulated in early stage ccRCC patients [41].

uEV-derived miR-204-5p is detected at high level in both 20- and 40-weeks-old Xp11 translocation RCC (tRCC) mice relative to control mice. This upregulated miR-204-5p is additionally observed in human Xp11 tRCC cell lines compared to normal cells, which is caused by overexpression of PRCC-TFE3 fusion gene. The comparable level of miR-204-5p in 20 and 40 weeks of age infers that uEVs can be biomarkers for early diagnosis of patients with Xp11.2 tRCC [43].

miR-224-5p is significantly upregulated in both uEVs and tissue from RCC patients compared to healthy controls. miR-224-5p stabilizes PD-L1 (programmed cell death protein 1) expression via directly suppressing the gene encoding cyclin D1 (*CCND1*). The study has elucidated the mechanism how miR-224-5p promotes resistance to T cell-dependent toxicity and metastasis via EVs transmission between RCC cells [44]. Cancer metastasis is the major cause of death of cancer patients and considered a hallmark of tumor progression. To invade, resist apoptosis, and disseminate, carcinoma cells must lose their epithelial phenotypes, detach from epithelial sheets, while gaining the mesenchymal characteristics. This reversible process called the epithelial–mesenchymal transition (EMT) which involves in-wound healing, embryogenesis and inflammation [53]. Podocytes and proximal tubular cell line HK-2 under renal damage condition develop EMT. In addition, these cells specifically exhibit elevated levels of miR-145 and miR-126 in EVs, in accordance with uEVs from diabetic nephropathy patients and lead to EMT progression [42].

The small RNA sequencing of uEVs of ccRCC patients shows significantly lower level of miR-30c-5p in ccRCC compared to healthy individuals. Indeed, this miR-30c-5p is the specific biomarker for RCC owing to its different expression between RCC patients and healthy controls, but it is not distinguishable in bladder and prostate cancer. The AUC, sensitivity and specificity of miR-30c-5p in the diagnosis of ccRCC are 0.8192 (95% confidence interval 0.7388–0.8996, *p* < 0.01), 68.57% and 100%, respectively. Indeed, miR-30c-5p directly binds and suppresses heat shock protein HSPA5 which promotes ccRCC progression [45].

Studies have shown that uEV-derived protein phosphorylation enabled to classify the grade difference of RCC [54]. A panel of uEV-derived proteins including CAIX, CP, MMP9, PODXL, DKK4, CD10, DPEP1, EMMPRIN, Syntenin 1 and AQP1 are new biomarker candidates for early stage of ccRCC [46]. Boccio et al. gained insights into the potential lipid biomarkers for RCC by analyzing uEVs from RCC patients. These cancer-derived EVs contain distinguished lipidome as phosphatidylinositols phosphate (PIP), lyso-phospholipids (Lyso), phosphatidylethanolamines (PE), phosphatidylcholines (PC), mono/di/three-glycerols (MG/DG/TG), phosphatidic acids (PA), gangliosides (GL), prostanoids (Pn) [47]. Furthermore, at the time of submitting this review, a clinical trial (NCT04053855) is expected to be completed in August 2024 which used electron microscopy and flow cytometry for percentage of CD9+/CA9+ uEVs in urine as ccRCC biomarker [55]. In summary, uEVs have shown the potential for optimal solution for less invasive, highly sensitive and specific diagnosis and prognosis of kidney cancer.

## Discussion

Urine diagnostics has limitations due to contamination with many factors and short-term stability of nucleic acids, but urine EVs and their contents retain high integrity in alternative temperature [39, 56–58]. Since EVs produced by the cells are membranous, the information is protected and accurate, which facilitates the application of uEVs in kidney cancer diagnosis and prognosis. To achieve a better outcome, combining EVs contents with other information would improve discrimination sensitivity and specificity between cancer patients and healthy participants. Even though a large amount of research has shown many potential markers, these biomarkers still need to be validated for clinical application. Further evaluation is required for the specificity of EVs related to kidney cancer since experimental models or sample sizes are limited. Other concern for optimizing the uEVs utilization in biomarker discovery for kidney cancer are normalization, quantification, and characterization in spot urines. There are several normalization approaches to compare uEV biomarkers among individuals such as urine creatinine, nephron mass or uEV excretion rate, total urine protein and albumin [37, 58, 59]. Despite these limitations, uEVs are a promising and applicable biomarker resource and can revolutionize clinical diagnosis, prognosis and treatment of kidney cancer patients in the future.
